# The impact of epidemics on labor market: identifying victims of the Middle East Respiratory Syndrome in the Korean labor market

**DOI:** 10.1186/s12939-016-0483-9

**Published:** 2016-12-01

**Authors:** Ayoung Lee, Joonmo Cho

**Affiliations:** 1 HRD Center, Sungkyunkwan University, 25-2 Sungkyunkwan-ro, Jongno-gu, Seoul 110-745 Republic of South Korea; 2 Department of Economics, Sungkyunkwan University, 25-2 Sungkyunkwan-ro, Jongno-gu, Seoul 110-745 Republic of South Korea

**Keywords:** Middle East Respiratory Syndrome, Quarantine, Vulnerability, Unemployment, Epidemics, Older workers, Labor Market

## Abstract

**Background:**

The vulnerability approach suggests that disasters such as epidemics have different effects according not only to physical vulnerability but also to economic class (status). This paper examines the effect of the Middle East Respiratory Syndrome epidemic on the labor market to investigate whether vulnerable groups become more vulnerable due to an interaction between the socio-economic structure and physical risk.

**Methods:**

This paper examines the effect of the Middle East Respiratory Syndrome epidemic on the labor market by considering unemployment status, job status, working hours, reason for unemployment and underemployment status. In particular, the study investigates whether the U-shaped curve becomes a J-shaped curve due to the interaction between medical vulnerability and labor market vulnerability after an outbreak, assuming that the relative vulnerability in the labor market by age shows a U curve with peaks for the young group and middle aged and old aged groups using the Economically Active Population Survey. We use the difference in difference approach and also conduct a falsification check and robustness check.

**Results:**

The results suggest that older workers faced a higher possibility of unemployment after the Middle East Respiratory Syndrome outbreak. In particular, they experienced higher involuntary unemployment and underemployment status as well as decreased working hours. It was confirmed that the relative vulnerability of the labor market for older workers was higher than for the other age groups after the epidemic outbreak due to the double whammy of vulnerability in the medical and labor market. The vulnerability in the young group partially increased compared to the 30s and 40s age groups due to their relative vulnerability in the labor market despite being healthy. We find that assuming the relative vulnerability in the existing labor market shows a U shape with age increase, the U-shaped curve became J-shaped after the outbreak.

**Conclusions:**

Disasters like epidemics can occur unexpectedly and affect certain groups more than other. Therefore, medical protection should be enhanced for groups vulnerable to disease and economic measures are also required for the protection of their livelihoods in the labor market to prevent unemployment stemming from inequality.

## Background

After an outbreak that lasted 172 days, the Middle East Respiratory Syndrome (MERS) epidemic was officially declared over on November 25, 2015 when the last MERS patient in Korea died. The Korean economy became sluggish for 6 months from the first MERS outbreak on May 20, 2015 and the Bank of Korea reduced the key rates from 1.75% down to 1.5% on June 11, 2015 [1]. This figure marked the lowest interest rates in Korean history [1]. The bank announced that the main reason for the interest rate cut was not only sluggish economic recovery but also a larger than expect impact from MERS on the economy, raising concerns about the worsened economic situation. Such events showed that MERS strongly affected the Korean economy [1].

About one in every 3000 Koreans was isolated due to MERS outbreak, making people psychologically unstable [2, 3]. Existing studies showed that an epidemic has behavioral effects (changes in behavior) due to the fear of contagion rather than direct effects (ex: labor supply decrease due to death), decreasing the demand for goods and services and lowering domestic income and employment [4, 5]. Therefore, it can be estimated that the impact of MERS on the Korean labor market was very large despite the low incidence and mortality rate.

Epidemics, a natural disaster, do not affect all people equally [6, 7]. It can be said that it is natural for epidemics to largely affect the groups that are physically vulnerable to diseases. Therefore, it is natural that the group with relatively high sensitivity to the epidemic receives the greatest negative impact compared to other groups. However, the vulnerability approach suggests that the effects of disasters like epidemics differ according to not only physical vulnerability but also economic class (status). The theory suggests that the vulnerable and peripheral groups in the dual labor market in Korea would be affected more by epidemics. Blaikie, Cannon, Davis and Wisner [8] stated that disaster affects people differently depending on the socio-economic pressure and the intersection with physical exposure. It can be estimated that disasters such as epidemics have a greater effect on vulnerable groups due to socio-economic conditions.

Disasters like epidemics can occur unexpectedly. The group that is relatively vulnerable in the labor market becomes more vulnerable due to the disaster if everyone is not equally affected and the group with vulnerability incurs more damages due to socio-economic conditions. Deeper gaps between groups can cause serious problems in labor markets suffering from polarization, as well as in Korean society as a whole. Therefore, economic measures are required to protect vulnerable groups in the labor market, as well as medical vulnerable groups in the event of disasters.

The purpose of this study is to investigate the effect of epidemics on the labor market. Specifically, the study focuses on whether the vulnerable class in the traditional labor market becomes more vulnerable due to the interaction between the medical and labor market vulnerabilities after an epidemic outbreak. Thus, the study examines various outcome in the labor market (unemployment status, job status (employed as temporary or permanent workers), hours worked, voluntary or involuntary unemployment and the underemployment status) to investigate whether a medically (physically) vulnerable group suffers a more adverse impact in the labor market after the MERS outbreak due to the structural vulnerability in the labor market.

### Related literatures

The vulnerability concept for disasters and natural hazards was introduced by O’Keefe, Westgate and Wisner in the 1970s [6]. In “Taking the naturalness out of natural disasters”, the authors claimed that the socio-economic condition was the cause of natural disasters. Natural disasters do not equally affect all people. Instead, the impact of the disaster is contingent on the vulnerability of the affected people, which often differs systematically across economic class, ethnicity, gender, and other factors [6]. In fact, a vulnerability approach to disasters would suggest that inequalities in exposure and sensitivity to risk as well as inequalities in access to opportunities systematically disadvantage certain groups of people, rendering them more vulnerable to the impact of natural disasters [7].

The risk-hazard model is a type of social vulnerability model. The Risk-Hazard (RH) model seeks to understand the impact of a hazard as a function of exposure to the hazardous event and the sensitivity of the entity exposed [9]. The Pressure and Release (PAR) model understands a disaster as the intersection between socio-economic pressure and physical exposure [8]. The vulnerability difference is caused by socio-economic conditions rather than the physical condition itself and such social vulnerability exacerbates the inequality.

The World Bank [5] reported that epidemics affect the economy through two channels. First is the temporary or permanent decrease in the labor supply due to the direct and indirect effects of sickness and mortality. Second is the behavioral effect due to people’s fear of contagion. Such behavioral effects cause a decrease in labor force participation due to the fear of contact with other people, shutting down places of employment. The Severe Acute Respiratory Syndrome (SARS) epidemic between 2002 and 2004 and the H1N1 flu epidemic in 2009 showed that the behavioral effects accounted for 80 or 90% of the impact on the economy [4, 5]. In addition, during the Ebola outbreak in 2014, the largest economic effects of the crisis was not the direct effect (mortality, morbidity, caregiving and the associated losses to working days), but the decrease in the goods and services demand by behavioral effects (changes in behavior) due to fear, reducing domestic income and employment. In particular, workers in service sectors like hotels and restaurants in Liberia were laid-off and the number of jobs was slashed by half [5].

An empirical study on actual epidemics by Fenichel [10] covers the effect of social distancing by epidemics on social welfare. The author states that social distancing and quarantine policies become ‘over-done’ and reduce social welfare. In particular, the social distance caused by epidemics may inhibit the epidemic spread but this phenomenon decreases the welfare of people that are not the targets. Also, it has an economically undesirable outcome and the health outcomes become potentially worse.

Eichelberger [11] stated that “The American public, including Chinatown, had become infected with an epidemic of fear, not of disease” during the SARS outbreak in 2003 even though there were only 8 people affected. Lee and Warner [12] examined the effect of SARS on the Hong Kong economy. In particular, the study on the labor market employment and unemployment level in the service sector (hotel) suggested that SARS sharply reduced the hotel occupation rates, increased underemployment and expanded no pay leaves.

Neumayer and Plümper [7] investigated whether socially vulnerable groups were affected more strongly by disasters than other groups. The analysis on 141 countries between 1981 and 2002 indicated that women faced a higher probability of death by natural disasters than men in countries where the socio-economic status (rights) of women was lower than that of men in the society. The authors reported that natural disasters had more adverse impacts on the vulnerable groups in the society and their result was consistent with the vulnerability approach.

### MERS and Korean labor market

MERS is an epidemic that spread the MERS-CoV in homes, hospitals and even through close contact between people. The symptoms of MERS included fever, cough, shortness of breath and myalgia for 98, 83, 72 and 32% of patients, respectively. Also, 26, 21 and 17% of patients suffered diarrhea, vomitting and abdominal pain, respectively and 72% of patients required mechanical ventilation. Assiri et al obtained data for all patients with laboratory-confirmed MERS-CoV infection from the Saudi Ministry of Health to WHO and for global MERS-CoV cases from ProMED, WHO, and CDC reports. They found MERS symptoms range from asymptomatic to severe pneumonia, leading to Acute Respiratory Distress Syndrome (ARDS) [13]. The medical charts of patients with confirmed MERS-CoV infection and household interviews with patients confirmed to have MERS-CoV infection by the Ministry of Health indicated that the impact was higher due to the psychological unfamiliarity with the disease, non-specific causes, and non-existence of a vaccine [14, 15]. The MERS virus was compared to the SARS and mentioned as a virus similar to SARS in initial reports.

The MERS break out started in Saudi Arabia in 2012 and expanded from the Arabian Peninsula. The World Health Organization [16] stated that 1599 people were infected, 574 people died and fatality reached 35.9%. A large outbreak later occurred in the Republic of Korea in 2015, resulting in the 2nd largest number of patients following Saudi Arabia [17].

**Fig. 1:**
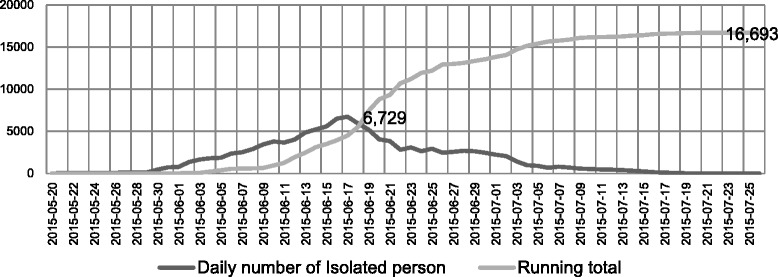
Status of the MERS isolated in Korea

The first case was found in a man who visited the Middle East on May 20, 2015. The number of people infected with MERS reached 186 and 38 died. 1164 schools were temporarily closed, and 16,752 people had been isolated as of November 20, 2015 (Fig. 1). This figure means that 1 out of every 3000 people in Korea was isolated. MERS severely shocked Korean society and scared people due to the lack of information and the perceived loss of control.

**Fig. 2:**
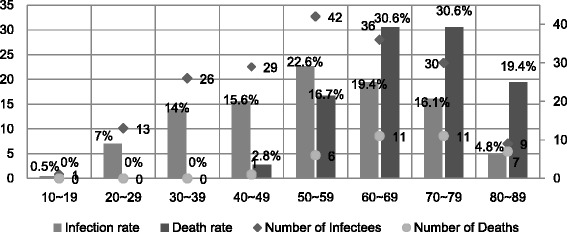
Deaths and infections from MERS in Korea by age groups

Figure 2 shows the number of deaths and infections by MERS according to age groups. MERS infection peaked among people in their 50s and the portion of patients was evenly distributed among the 30s, 40s, 60s and 70s age groups. Meanwhile, the number of deaths was high in the 60s and 70s age groups. Figure 2 implies that people in all age groups were afraid of the disease. It can be assumed that people aged over 50 were in the medically vulnerable group because the relative hazard rate in the middle aged and old groups were significantly higher than the other age groups [18].

MERS is an epidemic that spread through contact with people, and as a result leads to the avoidance of contact in people of all ages, especially causing older individuals with a high mortality rate to avoid contact. Such fear grows with the increase in social distancing [10], impacting the medical vulnerable group more significantly in social or economic aspects.

The effects of MERS mentioned above depend on the social structure [6, 7]. The vulnerability approach states that the effect not only from the physical aspect, but also the economic class (status) should not be ignored. The approach asserts that disasters like epidemics combine physical exposure to the epidemic with vulnerability due to structural problems in the economic system, resulting in a larger impact on certain individuals.

Organization for Economic Cooperation and Development [19] states that the young, women, middle aged and old people as well as migrants are vulnerable groups in the labor market. More than half of waged workers among young, middle aged and old people in Korea are non-regular workers [20]. Non-regular workers in Korea face highly job insecurity and low wages. Monthly average wages for non-regular workers remain half that of regular workers. Even worse, most regular workers receive national pension, health insurance and employment insurance from their companies as social insurance benefits but irregular works receive only 36.6, 37.7 and 34.5% of these benefits, respectively [20]. Generally, the Korean labor market shows a strongly stratified structure in which the portion of non-regular jobs with low wages and poor working conditions compared to standard jobs is high and exacerbates the inequality in the labor market [21, 22]. Therefore, the young, middle and old aged groups face a higher possibility of becoming more vulnerable than other groups in the Korean labor market system with the dual structure. It may be assumed that these people are in the labor market vulnerable group.

## Methods

### Conceptual framework and empirical strategy

**Fig. 3:**
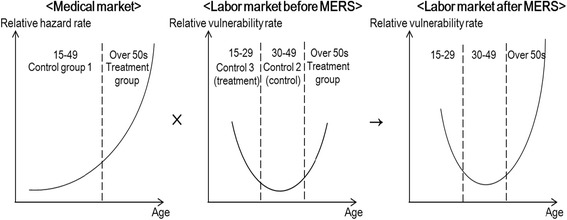
Relative vulnerability of medical and labor market

Figure 3 shows the intuitive relation between the relative vulnerability in the medical and labor markets and epidemics based on existing studies. It is assumed that medical vulnerability shows a concave increase with age and the relative vulnerability in the traditional labor market has a U shape with peaks in the youth, middle aged and old groups. The study investigates whether the U-shaped curve becomes J-shaped with a peak in the middle aged and old groups due to the interaction between the medical and labor market vulnerabilities after the outbreak.

We use the following difference in difference (DD) approach:

1 $$ {y}_{it}={\beta}_0+{\beta}_1pos{t}_t+{\beta}_2 treate{d}_{it}+{\beta}_3\left(pos{t}_t\times treate{d}_{it}\right)+{\beta}_4{X}_{it}+{\varepsilon}_{it} $$

where *y*_*it*_ is the outcome for person *i* at time *t*, *post*_*t*_ denotes a dummy variable that equals one if the observation is from June 2015 to July 2015 (after MERS), *treated*_*it*_ equals one if *i* is in the treatment group, and *X*_*it*_ denotes a vector of individual characteristics that include gender, educational attainment, marital status, and living area (urban). In our job status equations, *X*_*it*_ also contains occupation and industry variables. In log hours worked and underemployment status equation, *X*_*it*_ also contains job status (employed as temporary or permanent worker). The parameter *β*_3_ provides the estimated effect of MERS on the labor market in the treatment group relative to the control group. To ensure that the labor market outcome follows a similar trend and to control for seasonal variation in treated and control groups prior to the MERS outbreak, we restrict our main analysis to a time window incorporating the same 2 months in the previous year and 2 month after the MERS outbreak (i.e., June to July 2014 and June to July 2015). Also, the study performed additional analysis with the main analysis period for the same 2 months in 2013 and 2 months after MERS (i.e., June to July, 2013 and June to July, 2015). Because Sewol Ferry disaster happened in April 16, 2014 in Korea. It was one of the most serious human-made disasters with 291 deaths and 13 missing individuals as of June 8, 2014. The disaster caused sadness nationwide and caused sluggish demand and employment in the accommodation, restaurant, arts, sports and recreation sectors [23].

The age of subjects ranged between 15 and 70 years old, and the lower limit in the study was 15, which is the minimum age for work according to labor law. The high limit was 70, which is the actual age of retirement stated in the OECD [19]. The treatment and control groups are specifically defined in Fig. 3 to examine whether the U curves changes to a J curve in the study. The treatment group is that of people over 50 who are vulnerable to the medical and labor markets. It was necessary to separate the 50s from the 60s group because the latter is part of the officially retired group. Therefore, the study set up treatment groups for people in their 50s and the over 50s. First, the basic control group consisted of people aged between 15 and 49, who are relatively less vulnerable in the medical market, to investigate whether the medically vulnerable group experiences a negative impact in the labor market compared to the other groups. Second, the study set up another control group of people aged between 30 and 49 not vulnerable in the medical and labor markets to investigate whether the middle aged and old groups experience more adverse effects compared to the control group. Third, the study set up another control group of people aged between 15 and 29, vulnerable in the labor market like the treatment group but not in the medical market. The similarity between the treatment and control groups was increased to examine whether the consciousness of the medically vulnerable group interacts with the vulnerability in the labor market to cause more adverse effects. Lastly, the study set up a control group of people aged between 30 and 49 and a treatment group of people aged between 15 and 29 to additionally investigate whether the impact increases due to vulnerability in the labor market despite good health.

It is very important to compare outcome changes between treated and control groups and to set up groups to use the DD approach. First, the treatment is assumed to be exogenous. The epidemic is suspended due to unexpectancy. Also, the DD approach assumes a common trend between the treatment and control groups before the event. Therefore, the study examines this (falsification check), incorrectly setting the treatment year to a year prior to the MERS epidemic while referring to Ratcliffe and Scholder [24]. The study defines MERS as occurring in the years prior to 2015 for the falsification check. For example, it is defined that the epidemic event occurred in June 2011 instead of June 2015 to perform an analysis during the pre-treatment period of June to July 2010 and the post-treatment period of June to July 2011. We specify treatment years as 2011–2014, each including data from 2 months of the previous year pre-treatment and 2 months post-treatment. No effect may be expected from the falsification check if the common trend hypothesis is satisfied. Otherwise, the study performs a robustness check, which controls differential trends [24, 25]. We rerun the DD analysis, additionally including linear time trends for treated and control groups.

2 $$ {y}_{it}={\beta}_0+{\beta}_1pos{t}_t+{\beta}_2 treate{d}_{it}+{\beta}_3\left(pos{t}_t\times treate{d}_{it}\right)+{\beta}_4 tren{d}_t+{\beta}_5\left( treate{d}_{it}\times tren{d}_t\right)+{\beta}_6{X}_{it}+{\varepsilon}_{it} $$

*trend*_*t*_ is a time trend (starting from June 2010) to account for any differential trends in the labor market outcome prior to the MERS epidemic. The reason for setting the starting point of the data as 2010 for the time trend is to ensure an analysis period without the effect of the global crisis in 2008. We examine the effects of MERS on the labor market using data from pre-treatment years (i.e., from June 2010) to include monthly time trends (2010 m06, 2010 m07, 2011 m06,…).

### Data

The data used in the study is the Economically Active Population Survey (EAPS), the official monthly labor force survey of Korea. The EAP is the most widely used micro-level labor survey that provides basic information on unemployment on Korea [26]. It is similar to general labor force surveys in other countries, represents the Korean labor market with a sample of 32,000 households in Korea (about 70,000 individuals) and contains individual employment status in the week before the survey as well as other demographic characteristics. Using the sample weights, raw data are inflated to reflect the relevant population as well as to avoid sampling differences across different waves of the EAPS.

Appendix 1 shows descriptive statistics for the different definitions of treated groups and control groups in the analysis period. The results indicate that the portion of people employed as temporary workers is relatively high in the vulnerable group and that the young, middle aged and old aged groups are relatively vulnerable compared to people in their 30s and 40s in the labor market. It is also found that the probability of unemployment increased in the two groups after the MERS epidemic. The result does not show a large difference compared to 2014 but the probability of the unemployment largely increased for the groups compared to 2013. It may be inferred that the labor market was temporarily shocked due to the Sewol Ferry disaster in 2014. In addition, the working hours for the middle aged, old aged, 30s and 40s groups decreased slightly but increased slightly in the young group.

**Fig. 4:**
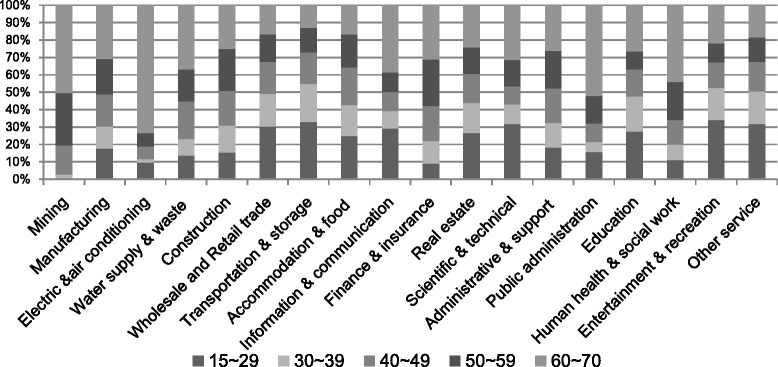
Relative ratio of temporary jobs for each industry

Figure 4 shows the portion of temporary workers in each industry. In particular, the study specifically investigated the portion of temporary workers for each age group in the industries affected by MERS. The production trend for industries in the industrial activity report of June, 2015 issued by the National Statistics Office states that the accommodation and food sectors recorded the largest negative production compared to the same month in the last year (-9.9%), followed by entertainment and recreation (-8.6%), publishing, communication and information (-6.3%), transportation and storage (-2.4%), wholesale and retail (-1.6%) and electricity and air conditioning (-0.9%). This suggests that the accommodation and food, entertainment and recreation, publishing, communication and information industries were significantly affected by MERS. Previous studies have indicated that the accommodation, restaurant and recreation sectors are affected the most by epidemics [5, 12]. In addition, the portion of temporary employment for young, middle aged and old groups is very high in the publishing, communication and information industries compared to other industries. Other affected industries show that the portion of temporary jobs for these age groups is high and they are in the peripheral sector of the labor market. Therefore, it may be assumed that MERS caused larger damage to vulnerable groups in the labor market.

## Results

Table 1 shows the effect of MERS on the unemployment status, employment position and working hours. First, the study investigates whether the medical vulnerable group of people over 50s experienced adverse effects in the labor market compared to their counterparts aged between 15 and 49. The unemployment status shows that the probability of unemployment for over 50s increased by about 17.18% compared to the other groups, ceteris paribus. The analysis with the pre-treatment period of June and July 2013 shows that the probability of unemployment for people over 50 increased to about 24.66% compared to people aged between 15 and 49. The additional analysis with the treatment group only for 50s shows the probabilities of 14.86% and 27.63% for workers in their 50s to become unemployed after the MERS outbreak. The probability of temporary employment increased by 1.22% compared to 2014, but decreased by 1.66% compared to 2013. In the treatment group in their 50s, the probability increased by 2.37% from 2014 but decreased by 1.60% against 2013. The effect of MERS on number of working hours per week showed that the working hours for people over 50s decreased by 1.88% compared to people aged between 15 and 49. It was found that the working hours for the 50s treatment group decreased by 2.27% compared to workers aged between 15 and 49. This means that the medically vulnerable group faced a high possibility of unemployment in the labor market and their working hours decreased compared to their counterparts. It seems desirable to investigate the falsification and robustness check first and then perform an analysis on the possibility of temporary employment.

**Table 1:** Difference-in-difference estimates for logistic and OLS regressions

Control	Treat		Unemployment status		Employed as temporary		Log hours worked	
			Odds ratio		Odds ratio		Coefficient	
			2014,6 ~ 7	2013,6 ~ 7	2014,6 ~ 7	2013,6 ~ 7	2014,6 ~ 7	2013,6 ~ 7
15–49	Over 50	Treated × post	1.1718^b^ (0.0030)	1.2466^b^ (0.0033)	1.0122^b^ (0.0013)	0.9834^b^ (0.0012)	-0.0188^b^ (0.0002)	-0.0171^b^ (0.0002)
	50s	Treated × post	1.1486^b^ (0.0034)	1.2763^b^ (0.0040)	1.0237^b^ (0.0015)	0.9840^b^ (0.0014)	-0.0227^b^ (0.0002)	-0.0164^b^ (0.0002)
30–49	Over 50	Treated × post	1.2069^b^ (0.0034)	1.3433^b^ (0.0039)	0.9795^b^ (0.0013)	0.9375^b^ (0.0012)	-0.0180^b^ (0.0002)	-0.0188^b^ (0.0002)
	50s	Treated × post	1.0948^b^ (0.0024)	1.2252^b^ (0.0028)	0.9472^b^ (0.0011)	0.9118^b^ (0.0011)	-0.0103^b^ (0.0002)	-0.0138^b^ (0.0002)
15–29	Over 50	Treated × post	1.1391^b^ (0.0031)	1.1537^b^ (0.0034)	1.0789^b^ (0.0017)	1.0746^b^ (0.0017)	-0.0210^b^ (0.0003)	-0.0149^b^ (0.0003)
	50s	Treated × post	1.1180^b^ (0.0035)	1.1830^b^ (0.0039)	1.0925^b^ (0.0019)	1.0722^b^ (0.0018)	-0.0253^b^ (0.0003)	-0.0138^b^ (0.0003)

^a^ and ^b^ indicate that the estimate is significant at the 0.05 and 0.01 levels, respectively. Standard errors are reported in parentheses. The reference group for the unemployment status is the employed and the reference group for the employed as temporary workers is the employed as permanent worker. Periods in the table are the pre-treatment period. The post-treatment period for all the analyses is June - July, 2015

Second, the study set up a control group of people aged between 30 and 49 not vulnerable in the medical and labor markets to investigate whether the vulnerable group experienced a great adverse effect compared to the control group. The result indicated that compared to people in their 30s and 40s, the group over 50s faced a higher probability of unemployment with an increase of 20.69% (compared with 2014) and 34.33% (compared with 2013). The probability of unemployment in treated group only for 50s relative to control increased by 9.48 and 22.52% compared to 2014 and 2013, respectively. The probability of temporary employment decreased by 2.05% (compared with 2014) and 6.25% (compared with 2013) in treated group relative to control. The working hours for the over 50s age group decreased by 1.80% (compared with 2014) and 1.88% (compared with 2013) compared to workers in their 30s and 40s after the MERS outbreak. This suggests that the unemployment possibility is much higher in the vulnerable group than in the counterpart, the probability for the temporary employment decreases and working hours decrease.

The study increased the similarity between the treatment and control groups in the labor market and compared the control group aged between 15 and 29 to workers over 50s to investigate whether the recognition of medical vulnerability shows an intersection with vulnerability in the labor market, resulting in more adverse effects. The probability of unemployment drops a little in comparison to the previous control group but treated group faced unemployment by 13.91% (compared with 2014) and 15.37% (compared with 2013) relative to control after the MERS outbreak. It may be inferred that both groups are vulnerable in the labor market, but treated group received more negative effects in the market due to their vulnerability to the epidemic. Relative to the permanent employment, the probability of temporary employment was confirmed to increase for workers over 50s. The working hours for those over 50s decreased after the MERS outbreak compared to their counterparts. The analysis with the 50s treatment group shows a similar trend.

As a falsification analysis, we specify treatment years as 2011–2014, each including data from 2 months of the previous year pre-treatment and 2 months post-treatment (Appendix 2). The analysis to check the previous trend shows that all the years except 2013 indicate that the group of workers over 50s faced a high possibility for unemployment compared to the control group. However, it suggests that the probability of unemployment changed only from 0.8 to 1.0%. Moreover, for the 50s treatment group, the possibility for unemployment decreased except compared to 2014 when the Sewol Ferry disaster happened, raising the possibility for employment for the middle aged and old people. Therefore, the comparison to the result of the falsification check can assert that the middle aged and old aged people faced a higher possibility for unemployment after the MERS outbreak as shown in Table 1.

It also shows the increasing trend in temporary employment for workers over 50s excepting the analysis in 2014 after the Sewol ferry disaster. The result is in line with previous studies indicating that workers over 50s have more irregular and temporary employment with lower wages and poorer working environments due to the dual structure in the labor market of Korea [27, 28]. The observation that middle aged and older people have a relatively low probability of temporary employment after 2014 due to the Sewol ferry disaster and 2015 due to MERS may be interpreted as follows. The decrease in the possibility of temporary employment may be caused by a higher portion of permanent employment or a decrease in temporary employment while maintaining the proportion of permanent employment. A lot of media reports stated that companies hired fewer workers, as well as sluggish consumption due to the disease. In particular, industries with high proportions of temporary jobs like the restaurant, accommodation, publication and recreation sectors (Fig. 4) were largely impacted, leading to reduce temporary jobs. Therefore, it may be that the temporary jobs for middle aged and older individuals decreased due to MERS. Meanwhile, it is highly probable that temporary employment opportunities decreased due to MERS outbreak. The trend indicates that the number of working hours was decreasing even before the MERS outbreak. Therefore, it seems more desirable to investigate the results after controlling the time trend for the study. Table 2 shows the result considering the differential time trend mentioned in Appendix 2.

**Table 2:** Robustness check, differential time trends

Control	Treatment		Unemployment		Employed temporary		Log hours worked	
			Odds	S.E	Odds	S.E	Coef	S.E
15–49	Over 50	Treated × post	1.2201^b^	0.0032	0.9615^b^	0.0012	-0.0217^b^	0.0002
		post	1.0960^b^	0.0014	1.0414^b^	0.0007	0.0131^b^	0.0001
		Time	0.9999^b^	0.0027	0.9991^b^	0.0014	-0.0130^b^	0.0002
		Treated × time	0.9996^b^	0.0061	1.0002^b^	0.0029	0.0025^b^	0.0004
	50s	Treated × post	1.1820^b^	0.0036	0.9746^b^	0.0014	-0.0275^b^	0.0002
		post	1.0968^b^	0.0013	1.0408^b^	0.0007	0.0132^b^	0.0001
		Time	0.9999^b^	0.0027	0.9991^b^	0.0014	-0.0127^b^	0.0002
		Treated × time	0.9999^b^	0.0072	1.0001^b^	0.0033	0.0050^b^	0.0004
30–49	Over 50	Treated × post	1.2471^b^	0.0037	0.9148^b^	0.0013	-0.0188^b^	0.0002
		post	1.0733^b^	0.0019	1.1004^b^	0.0009	0.0094^b^	0.0001
		Time	0.9997^b^	0.0039	0.9990^b^	0.0018	-0.0103^b^	0.0002
		Treated × time	0.9998^b^	0.0067	1.0003^b^	0.0031	0.0006^b^	0.0004
	50s	Treated × post	1.0809^b^	0.0025	0.8861^b^	0.0011	-0.0090^b^	0.0002
		post	1.0739^b^	0.0019	1.0973^b^	0.0009	0.0112^b^	0.0001
		Time	0.9997^b^	0.0039	0.9990^b^	0.0018	-0.0109^b^	0.0002
		Treated × time	1.0003^b^	0.0052	1.0003^b^	0.0026	-0.0014^b^	0.0003
15–29	Over 50	Treated × post	1.2071^b^	0.0035	1.0713^b^	0.0017	-0.0262^b^	0.0003
		post	1.1112^b^	0.0019	0.9387^b^	0.0011	0.0184^b^	0.0002
		Time	1.0002^b^	0.0039	0.9994^b^	0.0025	-0.0171^b^	0.0004
		Treated × time	0.9993^b^	0.0067	1.0000^b^	0.0035	0.0052^b^	0.0064
	50s	Treated × post	1.1689^b^	0.0039	1.0823^b^	0.0019	-0.0326^b^	0.0003
		post	1.1134^b^	0.0019	0.9367^b^	0.0011	0.0192^b^	0.0002
		Time	1.0002^b^	0.0039	0.9994^b^	0.0025	-0.0168^b^	0.0004
		Treated × time	0.9996^b^	0.0078	0.9999^b^	0.0039	0.0079^b^	0.0006

^a^ and ^b^ indicate that the estimate is significant at the 0.05 and 0.01 levels, respectively. It is the reported value after multiplying 1000 with the standard errors in the time and treated time and multiplying 100 with the time coefficient of the log hours working time and treated time. The reference group for the unemployment status is the employed and the employment permanent is the reference for the employed temporary

Despite considering the differential time trend, the possibility for unemployment was relatively high for workers over 50s after the epidemic and it decreased for temporary workers, as well as working hours. The analysis of control groups for workers aged between 30 and 49 and 15 and 49 indicated that the workers over 50s faced an unemployment increase by up to 24% but the probability of temporary jobs decreased. Also, the working hours further decreased in the middle aged and old groups after the epidemic outbreak even after the time trend control. The result that vulnerable groups experienced more negative effects in the labor market shows that the middle aged and old groups had a higher possibility for unemployment than their counterparts and that their working hours decreased. In particular, the workers over 50s experienced decreased working hours by up to 3.26% on a weekly basis. It may be estimated that the recognition of the medically vulnerable group may serve as an adverse effect in the labor market. The result is similar to that in Table 1 even after the robustness check, indicating that MERS negatively affected the vulnerable group in the labor market and that the interaction between medical vulnerability and vulnerability in the labor market created further negative effects.

However, the result may be overestimated considering the negative effect of the voluntary selection of unemployment in the labor market during the outbreak because the middle aged and old groups are vulnerable to epidemics. Therefore, the study analyzed voluntary or involuntary unemployment as shown in Table 3. The result of multinomial logistic regression to investigate voluntary or involuntary unemployment shows that compared to the group aged between 15 and 49, the group over 50s shows a 4.79% probability of voluntary unemployment and a 5.12% probability of involuntary unemployment after the MERS outbreak. The probability considering time trend increased by 5.86 and 7.27%, respectively, showing a higher probability of involuntary unemployment. In particular, the analysis with the 50s treatment group confirms that the probability of involuntary unemployment increases further. The comparison of the youth group to the workers in their 50s shows that the probability of voluntary unemployment decreases but the probability of involuntary unemployment largely increases by 13.55%. The figure rises to 14.15% considering the differential time trend. This result may be because the recognition of the medical vulnerability interacts with the vulnerability in the labor market, resulting in more adverse effects in the labor market.

**Table 3:** Difference-in-difference estimates for multinomial logistic regression (unemployment reason)

Control group	Treatment group	Unemployment reason				
			Relative Risk Ratio Pre-treatment: 2014, 6 ~ 7		Relative Risk Ratio Time trends control	
			Voluntary	Involuntary	Voluntary	Involuntary
15–49	Over 50	Treated × post	1.0379^b^ (0.0017)	1.0512^b^ (0.0022)	1.0586^b^ (0.0017)	1.0727^b^ (0.0023)
		post			0.9518^b^ (0.0008)	1.0616^b^ (0.0015)
	50s	Treated × post	1.0067^b^ (0.0020)	1.1536^b^ (0.0029)	1.0490^b^ (0.0022)	1.1694^b^ (0.0030)
		post			0.9519^b^ (0.0008)	1.0623^b^ (0.0016)
30–49	Over 50	Treated × post	1.0410^b^ (0.0019)	1.0644^b^ (0.0026)	1.0263^b^ (0.0020)	1.0966^b^ (0.0027)
		post			0.9836^b^ (0.0013)	1.0388^b^ (0.0020)
	50s	Treated × post	1.0087^b^ (0.0016)	1.1001^b^ (0.0027)	0.9605^b^ (0.0015)	1.1193^b^ (0.0028)
		post			0.9849^b^ (0.0013)	1.0442^b^ (0.0020)
15–29	Over 50	Treated × post	1.0287^b^ (0.0018)	1.0363^b^ (0.0029)	1.0900^b^ (0.0020)	1.0468^b^ (0.0031)
		post			0.9241^b^ (0.0011)	1.0865^b^ (0.0027)
	50s	Treated × post	0.9973 (0.0022)	1.1355^b^ (0.0036)	1.0823^b^ (0.0024)	1.1415^b^ (0.0037)
		post			0.9239** (0.0011)	1.0862^b^ (0.0027)

^a^ and ^b^ indicate that the estimate is significant at the 0.05 and 0.01 levels, respectively. Standard errors are reported in parentheses. Reported values are relative risk ratio and the reference group is the employed. The unemployed due to personal affairs, family affairs, raising children, house affairs, physical and mental disorder, retirement, age and working condition complaints are defined as the voluntary unemployment and involuntary unemployment is defined as the loss of jobs due to company suspension, closure, early∙honorary retirement∙ layoff, being out of work or sluggish business

It could not be confirmed whether the decreased working hours was a negative impact from the epidemic outbreak. The MERS epidemic in Korea was the similar to the SARS and H1N1 flu epidemics reported by existing studies [4, 5] and are infected by the epidemic of fear with high isolation index rather than the infect of the disease itself. It can be speculated that people were unwilling to visit crowded places like subway stations, restaurants and recreational facilities, reducing goods and service demand and negatively affecting the labor market. The flexible decrease in the working hours may be a positive aspect in the MERS epidemic. Therefore, it is necessary to investigate whether workers with fewer working hours want to work more. Table 4 defines workers who wish to increase their working hours as underemployment status and details the impact of MERS on the underemployment status.

**Table 4:** Difference-in-difference estimates for logistic regression (underemployment status)

Control	Treatment	Underemployment status		
			Odds Ratio Pre-treatment: 2014, 6 ~ 7	Odds Ratio Time trends control
15–49	Over 50	Treated × post	1.2837^b^ (0.0044)	1.0171^b^ (0.0035)
		post		1.2647^b^ (0.0030)
	50s	Treated × post	1.3489^b^ (0.0055)	1.0752^b^ (0.0043)
		post		1.2612^b^ (0.0030)
30–49	Over 50	Treated × post	1.3374^b^ (0.0050)	1.0844^b^ (0.0041)
		post		1.1880^b^ (0.0033)
	50s	Treated × post	1.2955^b^ (0.0049)	1.1704^b^ (0.0045)
		post		1.1872^b^ (0.0033)
15–29	Over 50	Treated × post	1.1929^b^ (0.0059)	0.8797^b^ (0.0044)
		post		1.4629^b^ (0.0064)
	50s	Treated × post	1.2551^b^ (0.0068)	0.9322^b^ (0.0051)
		post		1.4573^b^ (0.0064)

^a^ and ^b^ indicate that the estimate is significant at the 0.05 and 0.01 levels, respectively. Standard errors are reported in parentheses. The underemployed status is defined as wanting to increase the number of working hours, or wanting to move to a different job with more working hours. Reported values are odds ratio and the reference group is ‘maintaining working hours’

The analysis shows that compared to workers aged between 15 and 49, the workers over 50s showed a 28.37% higher rate of underemployment. Compared to workers aged between 30 and 49, the probability of underemployment in workers over 50s increased by 33.74%. The analysis with the treatment group in the 50s showed a similar trend. The comparison of the young group with similar vulnerability in the labor market shows that the probability of extended working hours after the MERS outbreak increased by 20% for workers over 50s.

The analysis considering the differential time trend showed that the treatment group had a high probability of underemployment compared to control group. However, the same result does not appear in the comparison with the young group, indicating that the group is also vulnerable in the labor market.

MERS is known to spread by contact between people. This makes people avoid contact with others, severely impacting working status in jobs with high physical exposure to people. Table 5 specifically investigates the effect of the epidemic on the number of working hours depending on the exposure risk from the epidemic. Occupational Safety and Health Administration [29] classifies very high exposure risk, high exposure risk, medium exposure risk and lower exposure risk depending on the employees with high-frequency contact with the general population. Similarly, to investigate the effect of MERS on the number of working hours, the study classifies the professionals in the human health and social work industry sectors (doctor, nurse, etc.) with the highest exposure to patients as having a very high exposure risk. Next, the services and sales workers in the industries affected by MERS (see Data section) are classified as having a high exposure risk, other service and sales workers as having medium exposure risk and technical and manual workers as having lower exposure risk.

**Table 5:** Difference-in-difference estimates for OLS regression (exposure risk)

Control	Treatment	Log hours worked				
			Very high	high	medium	low
A. Pre-treatment period: 2014, 6 ~ 7						
15–49	Over 50	Treated × post	-0.0140^b^ (0.0008)	-0.0522^b^ (0.0007)	-0.0176^b^ (0.0006)	-0.0181^b^ (0.0002)
30–49	Over 50	Treated × post	-0.0215^b^ (0.0009)	-0.0550^b^ (0.0006)	-0.0091^b^ (0.0007)	-0.0139^b^ (0.0002)
15–29	Over 50	Treated × post	-0.0067^b^ (0.0009)	-0.0421^b^ (0.0009)	-0.0448^b^ (0.0011)	-0.0324^b^ (0.0004)
B. Differential time trends control						
15–49	Over 50	Treated × post	-0.0874^b^ (0.0010)	-0.0535^b^ (0.0008)	-0.0191^b^ (0.0008)	-0.0177^b^ (0.0003)
		post	0.0246^b^ (0.0003)	0.0279^b^ (0.0004)	0.0034^b^ (0.0004)	0.0095^b^ (0.0001)
30–49	Over 50	Treated × post	-0.0903^b^ (0.0011)	-0.0390^b^ (0.0007)	-0.0084^b^ (0.0008)	-0.0146^b^ (0.0003)
		post	0.0278^b^ (0.0004)	0.0096^b^ (0.0004)	-0.0088 (0.0005)	0.0061^b^ (0.0003)
15–29	Over 50	Treated × post	-0.0783^b^ (0.0011)	-0.0695^b^ (0.0010)	-0.0543^b^ (0.0013)	-0.0274^b^ (0.0004)
		post	0.0203^b^ (0.0005)	0.0465^b^ (0.0006)	0.0392^b^ (0.0010)	0.0195^b^ (0.0004)

^a^ and ^b^ indicate that the estimate is significant at the 0.05 and 0.01 levels, respectively. Standard errors are reported in parentheses

The result shows that middle aged and old workers in the high exposure risk group largely decreased the working hours after the MERS outbreak compared to other age groups. It also shows that working hours for the group decreased against other groups in the industries not directly affected by MERS. In particular, it was found that the working hours for the middle aged and old groups largely decreased even in jobs with little contact with people compared to the young group.

Considering the differential time trends, it showed that working hours for middle aged and old workers in the human health and social work industry decreased by about 10% after the epidemic. Also, the working hours for the middle aged and old groups decreased in jobs with less physical exposure risk compared to other groups. The weekly working hours decreased sharply compared to the young group. These results are in line with those from Fenichel [10], indicating that the fear of people with high social isolation was ‘over-done’ and decreased the working hours of vulnerable workers in working environments with low epidemic exposure risk. In particular, manual workers take high portion of the lower exposure risk and the working hour decrease had a direct negative impact for a living in the manual workers.

In addition, we compare the control group of people aged between 30 and 49 and the treatment group of people aged between 15 and 29 to investigate whether the impact increases due to vulnerability in the labor market despite good health (Appendix 3). The result shows that the unemployment rate in treatment group increased further, particularly the possibility for involuntary unemployment. Meanwhile, the working hours increased a little bit.

The analysis results may imply that the vulnerability in the middle aged and old aged groups became higher than for the other age groups after the epidemic outbreak. At the same time, the vulnerability in the young group partially increased compared to the 30s and 40s age groups. Therefore, assuming that the relative vulnerability in the existing labor market shows a U shape, the U-shaped curve becomes J-shaped with a peak in the middle aged and old aged groups after the outbreak.

## Discussion

Our findings reveal that compared to the age group between 15 and 49 years old with no vulnerability to the disease, the workers aged over 50s with disease vulnerability had a high probability of unemployment after the MERS outbreak, their working hours decreased, and the possibility of involuntary unemployment and underemployment increased. The analysis of workers over 50s vulnerable to both factors compared to their counterparts in the medical and labor markets indicates that the old group experienced more adverse effects in the labor market after the MERS outbreak. Next, the study examined whether the vulnerability of the young and middle aged and old aged groups in the labor market became worse due to the medical vulnerability using two comparative groups. The analysis indicates that the MERS outbreak increased the possibility for unemployment and temporary employment and sharply decreased working hours for the middle aged and old aged group compared to the young group. In particular, the working hours decreased even in jobs with low risk of exposure to the epidemic. Moreover, involuntary unemployment and underemployment showed a higher probability. Compared to the 30s and 40s groups, the young group had a high possibility for unemployment, especially involuntary unemployment after the MERS outbreak.

The results indicate that the relative vulnerability in the traditional labor market changes from a U-shaped curve to a J-shaped curve after disasters like MERS. The middle aged and old aged groups became more vulnerable compared to other age groups due to their relative vulnerability in the labor and medical markets. The young group was partly affected compared to the 30s and 40s age groups due to their relative vulnerability in the labor market despite being healthy. The 30s and 40s age groups showed mid-level vulnerability to the disease and is affected the least overall compared to other groups, having the lowest vulnerability in the labor market.

The MERS epidemic in Korea had negative effects on the labor market despite low prevalence and mortality rates. It can be concluded from the high isolation index that the labor market condition became worse due to the fear of contagion. This effect is in line with reports from Lee and Mckibbin [4] and the World Bank [5] indicating that the negative effect of epidemics on the economy comes from the behavioral effect rather than a direct effect.

The middle aged and old aged groups had a high likelihood of falling under the vulnerable group in the dual labor market and medical market similar to the results of the vulnerability approach, confirming a more adverse effect in the labor market. This is consistent with the reports from Blaikie, Cannon, Davis and Wisner [8] and Neumayer and Plumper [7] stating that the vulnerable group becomes more vulnerable due to the intersection between the socio-economic structure and physical exposure risk.

## Conclusions

Disasters such as epidemics may affect certain groups more than others. However, there is a saying that “Social vulnerability is partially the product of social inequalities [30]”. If a disaster (epidemic) adversely impacts vulnerable individuals, not through the disease itself but through the labor market structure and, in a broader sense, the socio-economic conditions, this would cause a serious problem in the society affected by polarization. In addition, when an infectious disease breaks out, its possible impact on a country’s labor market and on the economy can appear in the form of direct or indirect (spillovers) effects [5, 31]. In other words, vulnerable people’s behavioral changes include avoiding consumption and leisure activities for fear of contagion, which in turn results in a decline in the consumption of goods and services, ultimately leading to additional indirect damage in labor market [31]. The empirical analysis provides information that can be used to customize policies to support people that can be negatively impacted by the epidemics in order to respond against economic stress. Therefore, we need to provide medical protection to groups who are vulnerable to disease and economic measures are also required to protect their livelihoods in the labor market in order to prevent unemployment stemming from inequality.

The strength of this study is that investigated the effect that disasters, such as epidemics, can have on employment status, which directly influences peoples’ livelihoods. Although many studies have examined the economic impact of epidemics, few studies have focused on the effect of an infectious disease on the labor market. In particular, this study empirically identified the different effects that disasters, such as epidemics, produce not only due to physical vulnerability but also to economic class (the channel of the labor market). While we recognize there are limitations to this study, this study cannot narrow the sample by selecting the industry and regions that suffered severe losses from the contagious disease due to limitations of the available data. If the analysis of specific dimensions of industry and regions is further carries out using data, the results are expected to appear in a more remarkable way and to provide more useful information for the policy.

Disasters such as epidemics can unexpectedly occur, therefore, medical protection should be improved for groups who are vulnerable to disease, and economic measures are also required to protect their livelihoods in the labor market in order to prevent unemployment stemming from inequality.

## Appendix 1

**Table 6:** Descriptive Statistics for the Analysis Samples

	2010, 6 ~ 7 – 2014, 6 ~ 7		2013, 6 ~ 7		2014, 6 ~ 7		2015, 6 ~ 7	
	Mean	S.D	Mean	S.D	Mean	S.D	Mean	S.D
15 ~ 29								
Employed	0.402	0.490	0.394	0.489	0.404	0.491	0.409	0.492
Employed as permanent	0.225	0.417	0.224	0.417	0.232	0.422	0.239	0.426
Employed as temporary	0.158	0.365	0.152	0.359	0.155	0.362	0.153	0.360
Self employed	0.020	0.139	0.018	0.132	0.018	0.133	0.018	0.133
Unemployed	0.037	0.188	0.036	0.185	0.042	0.201	0.046	0.208
Out-of-labor force	0.552	0.497	0.561	0.496	0.544	0.498	0.535	0.499
Hours worked	17.607	22.906	16.951	22.328	17.378	22.471	17.559	22.388
30 ~ 49								
Employed	0.732	0.443	0.734	0.442	0.742	0.438	0.746	0.436
Employed as permanent	0.412	0.492	0.436	0.496	0.444	0.497	0.452	0.498
Employed as temporary	0.173	0.378	0.157	0.363	0.157	0.364	0.155	0.362
Self employed	0.147	0.354	0.142	0.349	0.141	0.348	0.139	0.346
Unemployed	0.020	0.141	0.020	0.139	0.021	0.143	0.021	0.145
Out-of-labor force	0.218	0.413	0.218	0.413	0.211	0.408	0.209	0.406
Hours worked	35.199	23.036	34.548	22.482	35.030	22.347	34.883	22.226
50 ~ 70								
Employed	0.593	0.491	0.606	0.489	0.610	0.488	0.619	0.486
Employed as permanent	0.187	0.390	0.199	0.399	0.213	0.409	0.225	0.418
Employed as temporary	0.183	0.387	0.186	0.389	0.181	0.385	0.184	0.388
Self employed	0.223	0.416	0.221	0.415	0.216	0.412	0.209	0.407
Unemployed	0.014	0.118	0.012	0.110	0.014	0.119	0.018	0.132
Out-of-labor force	0.339	0.474	0.331	0.470	0.326	0.469	0.318	0.466
Hours worked	29.638	25.367	29.783	24.928	30.059	24.791	29.702	24.448
Inflated sample size	379,765,964		76,648,387		77,108,352		77,798,315	
Raw sample size	579,626		113,265		109,759		107,629	

Data: Economically Active Population Survey (EAPS)

## Appendix 2

**Table 7:** Falsification analysis

Control	Treatment		2011, 6 ~ 7	2012, 6 ~ 7	2013, 6 ~ 7	2014, 6 ~ 7
A. Dependent variable: Unemployment status						
15–49	Over 50	Treated × post	1.0747^b^ (0.0030)	1.0333^b^ (0.0028)	0.7767^b^ (0.0022)	1.0649^b^ (0.0029)
	50s	Treated × post	0.9883^b^ (0.0033)	1.1150^b^ (0.0036)	0.8073^b^ (0.0026)	1.1134^b^ (0.0035)
30–49	Over 50	Treated × post	1.0588^b^ (0.0031)	1.0673^b^ (0.0032)	0.7870^b^ (0.0024)	1.1175^b^ (0.0033)
	50s	Treated × post	0.9811^b^ (0.0022)	1.0944^b^ (0.0026)	0.9608^b^ (0.0023)	1.1204^b^ (0.0026)
15–29	Over 50	Treated × post	1.0794^b^ (0.0032)	0.9898^b^ (0.0030)	0.7682^b^ (0.0023)	1.0087^b^ (0.0030)
	50s	Treated × post	0.9938^b^ (0.0035)	1.0672^b^ (0.0038)	0.7986^b^ (0.0028)	1.0545^b^ (0.0036)
B. Dependent variable: Employed as temporary worker						
15–49	Over 50	Treated × post	1.0531^b^ (0.0014)	1.0269^b^ (0.0013)	1.0388^b^ (0.0013)	0.9702^b^ (0.0012)
	50s	Treated × post	1.0550^b^ (0.0016)	1.0308^b^ (0.0015)	1.0130^b^ (0.0015)	0.9609^b^ (0.0014)
30–49	Over 50	Treated × post	1.0668^b^ (0.0015)	1.0310^b^ (0.0014)	1.0629^b^ (0.0014)	0.9576^b^ (0.0013)
	50s	Treated × post	1.0789^b^ (0.0012)	1.0257^b^ (0.0012)	1.0693^b^ (0.0012)	0.9595 (0.0011)
15–29	Over 50	Treated × post	1.0151^b^ (0.0016)	1.0140^b^ (0.0016)	0.9815^b^ (0.0015)	0.9966^b^ (0.0015)
	50s	Treated × post	1.0164^b^ (0.0018)	1.0200^b^ (0.0018)	0.9610^b^ (0.0016)	0.9833^b^ (0.0017)
C. Dependent variable: Log hours worked						
15–49	Over 50	Treated × post	-0.0129^b^ (0.0002)	0.0156^b^ (0.0002)	0.0004^a^ (0.0001)	0.0009^b^ (0.0001)
	50s	Treated × post	-0.0114^b^ (0.0002)	0.0206^b^ (0.0002)	-0.0006^b^ (0.0002)	0.0058^b^ (0.0001)
30–49	Over 50	Treated × post	-0.0134^b^ (0.0002)	0.0108^b^ (0.0002)	0.0008^b^ (0.0001)	-0.0014^b^ (0.0001)
	50s	Treated × post	-0.0067^b^ (0.0001)	0.0011^b^ (0.0001)	0.0011^b^ (0.0001)	-0.0039^b^ (0.0001)
15–29	Over 50	Treated × post	-0.0120^b^ (0.0003)	0.0235^b^ (0.0002)	-0.0015^b^ (0.0002)	0.0059^b^ (0.0002)
	50s	Treated × post	-0.0100^b^ (0.0003)	0.0283^b^ (0.0002)	-0.0028^b^ (0.0002)	0.0115^b^ (0.0002)

^a^ and ^b^ indicate that the estimate is significant at the 0.05 and 0.01 levels, respectively. Standard errors are reported in parentheses. The reference group for the unemployment status is the employed and the reference group for the employed as temporary worker is the employed as permanent worker. Reported values in panels A and B are odd ratios and panel C is the coefficient value

## Appendix 3

**Table 8:** Difference-in-difference estimates for logistic and OLS regressions (15-29 vs 30-49)

Control	Treatment		Unemployed	Employed temporary	Log hours worked	Unemployment reason	
						Voluntary	Involuntary
			Odds ratio	Odds ratio	Coef	RRR	RRR
A. Pre-treatment period: 2014, 6 ~ 7							
30–49	15–29	Treated × post	1.0595^b^ (0.0025)	0.9126^b^ (0.0013)	0.0022^b^ (0.0002)	1.0039^a^ (0.0017)	1.0265^b^ (0.0031)
B. Pre-treatment period: 2014, 6 ~ 7							
30–49	15–29	Treated × post	1.1660^b^ (0.0028)	0.8904^b^ (0.0013)	-0.0083^b^ (0.0002)	0.9190^b^ (0.0015)	1.2777^b^ (0.0039)
C. Differential time trends control							
		Treated × post	1.0351^b^ (0.0026)	0.8518^b^ (0.0012)	0.0073^b^ (0.0002)	0.9358^b^ (0.0016)	1.0432^b^ (0.0032)
		post	1.0762^b^ (0.0019)	1.1024^b^ (0.0009)	0.0116^b^ (0.0002)	0.9883^b^ (0.0013)	1.0429^b^ (0.0020)

^a^ and ^b^ indicate that the estimate is significant at the 0.05 and 0.01 levels, respectively. Standard errors are reported in parentheses. Reference group for unemployment status is employed, Employed and reference group for employed as temporary is employed as permanent

## Abbreviations

DD: Difference in difference
EAPS: Economically Active Population Survey
KLI: Korea Labor Institute
MERS: Middle East Respiratory Syndrome
OECD: Organization for Economic Cooperation and Development
OSHA: Occupational Safety and Health Administration
PAR: Pressure and Release
RH: Risk-Hazard
SARS: Severe Acute Respiratory Syndrome
WHO: World Health Organization
